# Patterns in Nuclear and Mitochondrial DNA Reveal Historical and Recent Isolation in the Black-Tailed Godwit (*Limosa limosa*)

**DOI:** 10.1371/journal.pone.0083949

**Published:** 2014-01-09

**Authors:** Krijn B. Trimbos, Camiel Doorenweerd, Ken Kraaijeveld, C. J. M. Musters, Niko M. Groen, Peter de Knijff, Theunis Piersma, Geert R. de Snoo

**Affiliations:** 1 Department of Conservation Biology, Institute of Environmental Sciences (CML), Leiden University, Leiden, The Netherlands; 2 Department of Terrestrial Zoology, Netherlands Centre for Biodiversity, Naturalis, Leiden, The Netherlands; 3 Department of Human Genetics, Leiden Genome Technology Center, Leiden University Medical Center, Leiden, The Netherlands; 4 Department of Animal Ecology, Center for Ecological and Evolutionary Studies, University of Groningen, Groningen, The Netherlands; 5 Department of Marine Ecology, NIOZ Royal Netherlands Institute for Sea Research, Den Burg, Texel, The Netherlands; University of Innsbruck, Austria

## Abstract

On the basis of morphological differences, three subspecies of Black-tailed Godwit (*Limosa limosa*) have been recognized (*L. l. limosa, L. l. islandica* and *L. l. melanuroides*). In previous studies mitochondrial DNA (mtDNA) sequence data showed minimal genetic divergence between the three subspecies and an absence of sub-structuring within *L. l. limosa*. Here, population genetic structure and phylogeographic patterns have been analyzed using COI, HVR1 and HVR2 mtDNA sequence data as well as 12 microsatellite loci (nuDNA). The nuDNA data suggest genetic differentiation between *L. l. limosa* from Sweden and The Netherlands, between *L. l. limosa* and *L. l. islandica*, but not between *L. l. limosa* and *L. l. melanuroides*. However, the mtDNA data were not consistent with the nuDNA pattern. mtDNA did support a split between *L. l. melanuroides* and *L. l. limosa*/*L. l. islandica* and also demonstrated two *L. l. limosa* haplotype clusters that were not geographically isolated. This genetic structure can be explained by a scenario of isolation of *L. l. melanuroides* from *L. l. limosa* in Beringia during the Last Glacial Maximum. During the Pleistocene separation of *L. l. islandica* from *L. l. limosa* occurred, followed by colonization of Iceland by the *L. l. islandica* during the Holocene. Within *L. l. limosa* founder events, followed by population expansion, took place during the Holocene also. According to the patterns observed in both markers together and their geographic separation, we propose that the three traditional subspecies indeed represent three separate genetic units.

## Introduction

Black-tailed Godwits are migratory shorebirds breeding mainly in temperate and boreal lowlands. Their breeding range extends across Eurasia, from Iceland to Kamchatka and Sakhalin [8]. Until a few centuries ago, breeding Black-tailed Godwits (*Limosa limosa* Linneaus, 1785) were confined to raised bogs, moorlands, lake margins and damp grassy depressions in steppe [2], [21]. Since the early Middle Ages the bog habitats in north-western Europe became converted into increasingly nutrient-rich meadows for dairy farming. Black-tailed Godwits were probably quick to exploit this new opportunity and as a result the number of breeding pairs in The Netherlands alone increased to approximately 120,000 in 1967 [35]. However, over the last few decades further agricultural intensification with increasingly early mowing dates has led to low recruitment [27], [50]. In addition, urbanization of rural areas has led to fragmentation of their breeding habitat. As a result, the mainland European breeding population has been in decline over the last 40 years [4], [50], [65]. This has prompted the IUCN to qualify the species as Near-Threatened [3].

**Figure 1:**
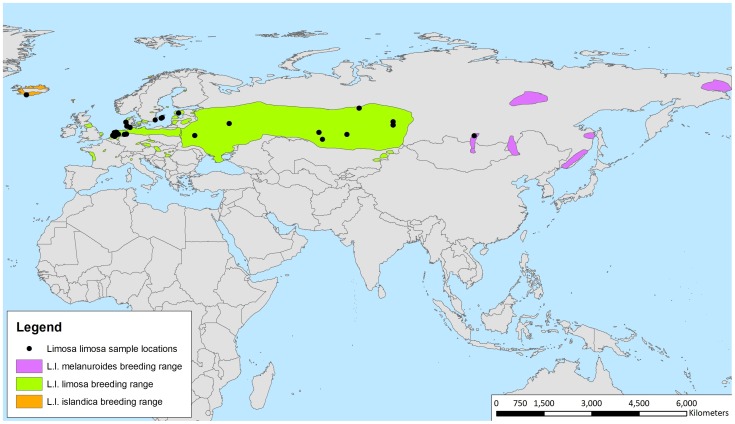
Sample locations of the Black-tailed Godwit *Limosa limosa*. Sample locations of the Black-tailed Godwit *Limosa limosa*. *L. l. limosa*: the Netherlands, Mid-Germany, Northern Germany, Denmark, Sweden, Belarus/Moscow, Kazachstan/SW Russia; *L. l. islandica*: Iceland; and *L. l. melanuroides*: Eastern Russia/Selanga delta.

Currently, three subspecies are recognized (Figure 1): the European Black-tailed Godwit (*Limosa limosa limosa*), Icelandic Black-tailed Godwit (*L. l. islandica*) and Asian Black-tailed Godwit (*L. l. melanuroides*) [8]. These subspecies have been distinguished on the basis of morphological traits. *L. l. islandica* has a shorter bill and tarsus and has more extensive rufous-cinnamon and barred plumage than *L. l. limosa*, while *L. l. melanuroides* is distinctly smaller compared to *L. l. limosa* [8],[44]. However, the phenotypic variation within and between different *Limosa* subspecies overlaps and varies throughout the seasons, often making it difficult to identify them with 100% certainty [31]. Aside from external characters, *Limosa* subspecies also differ in breeding range and migratory routes, although there is some overlap [17], [31]. The breeding range of *L. l. limosa* extents from Britain to West Russia. *L. l. islandica* breeds mainly on Iceland, with some breeding pairs occasionally found in Scotland and Northern Norway. *L. l. melanuroides* breeds at isolated locations in Russia, east of the Yenisey river. *L. l. limosa* winters in parts of southern Europe and south-west Asia, but mainly in sub-Saharan Africa. *L. l. islandica* migrates to Britain, western France, The Netherlands and Iberia. The wintering grounds of *L. l. melanuroides* are in south-east Asia, from the Bay of Bengal to Taiwan, the Philippines and Australia [8], [17].

Höglund *et al.* (2009) [23] found slight diagnostic differences between the subspecies on the basis of mitochondrial DNA (mtDNA) sequence data, but found no population structure within *L. l. limosa*. Although they had sequenced part of the highly variable control region (CR) of the mtDNA, they used a relatively conserved part in their analyses [29], [48]. This may have caused an underestimation of the genetic splits that are actually present between *L. l. limosa* populations. Using microsatellite markers targeting nuclear DNA (nuDNA), Trimbos *et al.* (2011) [57] found moderate levels of genetic variation among Black-tailed Godwits breeding in The Netherlands, and also did not detect any form of population structure. This suggests that: either fragmentation of Black-tailed Godwit breeding populations is too recent for lineage sorting to be complete, or gene flow has not been restricted on the scale of The Netherlands. However, genetic structure has yet to be studied in detail throughout the entire breeding range of the Black-tailed Godwit.

Owing to its four times smaller effective population size, mtDNA exhibits faster lineage sorting compared to nuDNA [34], [47], [64]. This difference in effective population size is attributed to the different ways in which the two genomes are inherited. Nuclear DNA is diploid, and recombined between both parents in every generation, whereas mtDNA is haploid and only inherited maternally. In theory, mtDNA could thus reflect changes in population structure faster. It has been argued, however, that the best measures of population genetic structure derive from the accumulated signals from multiple loci [10], whilst the entire mtDNA is effectively a single locus. With this in mind, we used a combination of both nuDNA and mtDNA data to account for the shortcomings of each [32], [46]. More specifically, to clarify population genetic structure of the Black-tailed Godwit in detail, genetic differentiation within *L. l. limosa* with respect to the divergence between the different *Limosa limosa* subspecies was studied using the mtDNA COI, HVR1 and HVR2 regions next to 12 nuDNA microsatellite loci.

## Materials and Methods

### Sample collection and DNA extraction

Samples were collected between 1991 and 2010 from sites across the *Limosa limosa* breeding range (Figure 1). Animal work in this study included taking blood of individual Black-tailed Godwits. Additionally, birds were colour ringed and biometrics were done for other research purposes. The animal work done here was approved by the Institutional Animal Care and Use Committee of the University of Groningen (IACUC-RuG). To limit stress, individual birds were handled for a maximum of 20 minutes. A blood sample of 20 ul was taken from the brachial wing vein before body size and plumage measurements were taken. The area around the vein was cleaned with a cotton ball dipped in ethanol. The blood was drawn from the puncture with a sterilized micro-capillary tube. The sample was stored in 96% ethanol at −20°C for the first weeks and at −80°C thereafter. Blood samples were taken in the field close to the nesting site so that the birds were handled in the most comfortable environment. Blood samples were taken at the beginning of the work to ensure that bleeding had stopped when all the work on the bird was finished and the birds could fly back to their nesting site instantly. Birds that expressed signs of high stress levels (fast panting, leg cramps) were freed immediately. Since the Black-tailed Godwit is a protected species exemption was needed and obtained from the Dutch Flora & Fauna act article 75 and the Dutch Animal Welfare Act article 9. Most blood samples were collected in The Netherlands. Other blood samples, previously collected (in Sweden, Russia/Moscow, Kazakhstan, western Russia, Iceland, Eastern Russia/Selenga Delta and Canada) by Höglund *et al.* (2009) [23], were made available by the University of Groningen, where they were stored (Table 1). Permissions to catch Black-tailed Godwits, collect egg shells and take blood in reserves were obtained from the appropriate authority in this case Staatsbosbeheer and It Fryske Gea. The rest of the sample collection was done on private land where we got permission of the different owners to conduct our studies.

**Table 1:** Geographical and genetic information of the used samples.

Region	Sample location	nuDNA	HVR	COI	Limosa species/subspecies
Netherlands (140)	Eemnespolder/Arkemheen	24	6	2	*Limosa limosa limosa*
	Grote Zoeterwoudse polder	11	4	1	*Limosa limosa limosa*
	Vijfheerenlanden	10	4	3	*Limosa limosa limosa*
	Uitdam	11	3	1	*Limosa limosa limosa*
	Polder Zeevang	11	4	2	*Limosa limosa limosa*
	Normerpolder	7	4	4	*Limosa limosa limosa*
	Overijssel/Zwolle	10	4	4	*Limosa limosa limosa*
	South-west Frysland	38	12	7	*Limosa limosa limosa*
	Vechtplassen	2	1	1	*Limosa limosa limosa*
	Idzegea	18	4	0	*Limosa limosa limosa*
Germany (35)	Mid-Germany, Schneckenbruch	3	2	2	*Limosa limosa limosa*
	Mid-Germany, Dummer	20	7	6	*Limosa limosa limosa*
	Northern Germany, Fohr	11	3	3	*Limosa limosa limosa*
	Northern Germany, Meggerdorf	1	1	1	*Limosa limosa limosa*
Denmark (11)	Tipperne	11	4	3	*Limosa limosa limosa*
Belarus (6)	Belarus	3	3	3	*Limosa limosa limosa*
	Moscow	3	3	1	*Limosa limosa limosa*
Sweden (42)	Kristianstad/Faludden/Hummelbosholm/Oland	42	4	2	*Limosa limosa limosa*
Kazakhstan, SW Russia (23)	Novosibirsk	5	2	0	*Limosa limosa limosa*
	Lake Ubinsky	2	0	0	*Limosa limosa limosa*
	Lake Sharkol	5	2	1	*Limosa limosa limosa*
	Lake Baituma	2	1	0	*Limosa limosa limosa*
	Lake Big Aksuhat	1	0	0	*Limosa limosa limosa*
	Lake Shoskaly	2	2	2	*Limosa limosa limosa*
	Juganski	6	1	2	*Limosa limosa limosa*
Iceland (27)	W. Iceland	27	5	3	*Limosa limosa islandica*
Eastern Russia (3)	River Selenga Delta	3	3	1	*Limosa melanuroides*
Canada (2)	Churchill, Manitoba	2	2	1	*Limosa heamastica*

Region, Sample location, number of samples per sample location used for microsatellite analysis (nuDNA), number of samples per sample location used for HVR mtDNA analysis (HVR), number of samples per sample location used for COI mtDNA analysis (COI) and the *Limosa* species or *Limosa limosa* subspecies per sample location.

Additionally, eggshells were obtained between 2008 and 2010 [56] in The Netherlands, Germany, Belarus and Denmark, all breeding areas of *L. l. limosa* (Table 1). For the collection of egg shell membranes no Black-tailed Godwit individuals were handled. Eggshell remains were collected in the nest (after hatching) and were individually stored in plastic bags at room temperature. DNA was extracted from 6–10 µl of blood using ammonium acetate [43] or the Qiagen DNeasy Blood and Tissue Kit according to the manufacturer's protocol [40]. DNA from eggshell membranes was also extracted using the Qiagen DNeasy Blood and Tissue Kit [40], with minor modifications as described by Trimbos *et al.* (2009) [56]. Publicly available sequences from the Barcoding of Life Database (BOLD) were used to supplement the COI barcodes and to provide an outgroup for the COI tree. The Hudsonian Godwit *Limosa haemastica*, an arctic-breeding godwit of Canada and Alaska, was used as outgroup for the HVR analysis.

### Microsatellite analysis

For the nuDNA data we used a set of microsatellite markers [60] which were previously utilized in Trimbos *et al.* (2011) [57]. A total of 289 birds from 10 different breeding locations were genotyped at 12 microsatellite loci. These 12 loci (LIM3, LIM5, LIM8, LIM10, LIM11, LIM12a, LIM24, LIM25, LIM26, LIM30, LIM33) were specifically developed for Black-tailed Godwits [60]. A Fisher's exact test for linkage disequilibrium was carried out using all 289 samples, with 1,000 dememorization steps, 100 batches and 1,000 iterations per batch (GENEPOP web version 4.0; [41]). Deviations from Hardy-Weinberg equilibrium and heterozygote excess or deficiency were tested for each locus and sampling location separately using 1,000 dememorization steps, 100 batches and 1,000 iterations per batch (GENEPOP; [41]). Bonferroni correction for multiple testing was applied [42]. To detect scoring and amplification errors, we employed MICRO-CHECKER with a 95% confidence interval over 10,000 runs [59].

For each location, observed (H_o_) and expected (H_e_) heterozygosities and inbreeding values (F_IS_) were estimated using ARLEQUIN 3.11 [13] set at 20,000 permutations. An analyses of molecular variance (AMOVA) was performed, allowing variance among sample locations (V_a_), variance within sample locations (V_b_) and residual variance to be computed (V_c_), using ARLEQUIN with 20,000 permutations, followed by Bonferroni correction. Additionally, D was calculated with 10,000 bootstraps using SPADE [7], as recent studies have indicated that this statistic provides more accurate estimates of genetic differentiation than F_ST_ [25], [33]. The number of private alleles was determined using CONVERT 1.31 [18]. FSTAT 2.9.3.2 [19] was used to calculate allelic range, number of alleles per sample location and allelic richness per sample location. To correct for sample size, this program uses the rarefaction index.

STRUCTURE 2.3.1 [39] was used to cluster genotypes from all sampling locations. We determined the deltaK (Structure Harvester), a calculation of the second-order rate of change in log likelihood Ln P(X|K), as recommended by Evanno *et al.* (2005) [12]. The most likely number of genetic clusters (K) in our sample set was also investigated by determining the maximum average log likelihood Ln P(X|K). Values computed with both methods were plotted using Structure Harvester 0.56.3 [9]. The Structure model was run using admixture and correlated allele frequencies. Additionally, the LOCPRIOR model, incorporated into STRUCTURE 2.3.1, was used. This model assumes that individuals sampled close together are often from the same population and can assist in the clustering when population structure is weak. To choose an appropriate burn-in length, we used the values of summary α statistics that are printed out by the program to see whether they appeared to had converged. The program was initially run 5 times with a burn-in period of 200,000 iterations and a length of 1,000,000 MCMC iterations for K (1–13) for the entire dataset. Additionally, STRUCTURE was run, 10 times with a burn-in of 500,000 and a length of 1,000,000 MCMC iterations for K(1–10), for the dataset without the *Limosa heamastica* samples, to make sure that the genetic signal of the *Limosa heamastica* would not bias the outcome of the STRUCTURE analysis. Since both datasets gave the same picture, we chose to show the STRUCTURE picture of the entire dataset here.

Convergence was assessed by checking whether the alpha graphs provided by the program reached equilibrium before the end of the burn-in phase. CLUMPP was used to estimate the number of identical repeat runs per K. The output of CLUMPP was accordingly used to generate graphs from the STRUCTURE results using Microsoft Excel.

A Mantel test with 9999 permutations was performed using GENALEX 6.2 to test for correlation between the genetic and geographic distance matrices [38].

### Mitochondrial DNA sequencing

We first sequenced part of the mitochondrial Cytochrome C Oxidase I (COI) gene, for a subset of samples. There is a large and growing database of COI barcodes [5], including barcodes for many bird species [51]. COI data allowed for easy comparison of the results from our samples with those of other studies. Secondly, we used next-generation sequencing on the Illumina HiSeq platform to determine primer sites for the amplification of the hypervariable regions HVR1 and HVR2 of the mitochondrial control region (CR). To identify suitable primer sites around the hypervariable sites (HVR1 and HVR2) in the control region of the mtDNA, we sequenced the entire mtDNA of three *L. l. limosa* samples (from The Netherlands, Sweden and SW Russia) at low coverage. For each sample, 1000 ng of genomic DNA was sheared to 500 bp fragments using a Covaris S2. These fragments were end-repaired and fitted with an A-overhang at the 3′ end using NEBNext TruSeq. Adapters were ligated to these fragments, after which they were sequenced on an Illumina HiSeq2000. The resulting reads were aligned against the complete mitochondrial sequence of the Ruddy Turnstone *Arenaria interpres* [37] using Stampy [58]. Barcoded DNA pools sequenced on part of a single lane of an Illumina HiSeq resulted in 817,335, 6,804,981 and 3,273,078 paired-end reads from *L. limosa* samples from the Netherlands, Sweden and SW Russia, respectively. Alignment of the Illumina reads to the *A. interpres* mtDNA with the substitution rate set to 0.1 resulted in 982, 10,068 and 806 aligned reads, respectively. These covered the mtDNA genome 0.58, 9.37 and 2.6 times, respectively. A consensus sequence was constructed using Samtools pileup [49]. On the basis of this consensus sequence, primers were developed amplifying the first and third domain of the *L. limosa* CR (5′-3′; F-primer: L13F 16650 – AGCAGTTCCTGCTTGGCTTT, R-primer: L13R 465 – GCAAGTTGTGCTAGGGGTTT and 5′-3′; F-primer: L23F 749 – TTCAAGTGTCCGGGGAATCA, R-primer: L23R 1225 –TTTGTCTCTGGGTGCATGGG). As sequencing with L13F and L23R proved to be problematic owing to long T-trains and CAAACAAAA repeats, further sequencing was performed unidirectional using only primers L13R and L23F. For HVR1 and HVR2, 649 bp were sequenced in 91 samples, including 81 *L. limosa* individuals from 23 different *L. l. limosa* breeding locations, five *L. l. islandica* individuals from Iceland, three individuals *L. l. melanuroides* from Eastern Russia and two *L. haemastica* individuals (Table 1). However, for other HVR1 and HVR2 analysis five sequences of poor quality, including the two samples from *L. haemastica* were excluded, adding up to a sample set of 78 samples from *L. l. limosa* breeding locations, five *L. l. islandica* from Iceland and three *L. l. melanuroides* from Eastern Russia.

The universal COI mitochondrial barcode region was amplified using primers BirdF1, BirdR1 and BirdR2 with the addition of M13 tails [22]. A cocktail of all three primers was used to increase PCR success rate. A section of 658 bp of the COI gene was sequenced for a subset of 56 samples, which included 52 individuals from several *L. l. limosa* breeding locations, three *L. l. islandica* from Iceland and one *L. l. melanuroides* from Eastern Russia.

PCR amplification reactions for L13 and L23 primer pairs were carried out in a total volume of 25 µl consisting of 10 ng genomic DNA, 2.5 µl PCR Buffer 10× including 15 mM MgCl_2_, 2.5 mM dNTP, 110 pmol of each primer, 1.25 U Taq DNA polymerase (Qiagen) and 18.8 µl DNA mQ water. For COI the same volume and PCR mix reagents were used with the exception of the amount of primer, which was now 250 pmol of each primer (M13F-BirdF1, M13R-BirdR1 and M13R-BirdR2). PCR was conducted on a BIORAD S1000 thermal cycler using the following PCR program: 94°C for 3 min; 40 cycles of 94°C for 15 s, locus-specific Ta 30 s, 72°C 40 s; 72°C for 5 min. Ta was 50°C for COI and 58°C for L13 and L23. With each PCR a negative control was included and sequenced to check for contamination issues. Sequencing was outsourced to Macrogen Europe. Forward and Reverse chromatograms were combined in Sequencer v4.10.1 (Gene Codes Corporation), checked manually for ambiguities, exported as FASTA files and aligned using BioEdit v7.0.9 [20]. All novel sequences generated for this study are deposited at GenBank (accession numbers JQ657268-JQ657500). The COI fragments were checked for NUMTs by examining chromatograms for double signal and by translating all fragments into amino acids and making sure there were no stop-codons, which would indicate a non-functional gene.

### Mitochondrial DNA analysis

For the mtDNA the number of haplotypes, haplotype diversity were calculated using dnaSP v5.0 [28], with gaps excluded as potential sequence variability. For HVR the number of indels and variable sites were given additionally. To detect past population expansions we calculated Fu's F_S_ statistic and Tajima's D-test [15], [54]. To test for background selection Fu and Li's D* and F* statistics were used [16]. To obtain pairwise Φst between sampling sites, pairwise Juke and Cantor distances and haplotype frequencies were calculated in ARLEQUIN 3.11 [13] with 20,000 permutations. A median-joining haplotype network was constructed using NETWORK v. 4600 (Fluxus-engineering).

DNA barcodes are available for 91% of all bird species [51], allowing for a comparison of the genetic variation of the mtDNA within *Limosa limosa* with other bird species [26], [51]. As DNA barcoding aims to identify species, the BOLD data structure does not recognize subspecies. However, subspecies clusters were recognized nonetheless through our own added subspecies COI sequence data and comments in the ‘notes’ field in some BOLD records. Some of the BOLD specimens had accompanying museum voucher pictures within the BOLD database. While these voucher pictures in theory can be used to determine if the plumage fits the designated subspecies, this was of little use in these cases as the voucher pictures did not show the correct profile to do this adequately. Phylogenetic analysis of the mtDNA was performed using maximum likelihood analysis. For the HVR tree *L. haemastica* (CAN) was used as an outgroup and for the COI tree public sequences of *Limnodromus scolopaceus* and *Limnodromus griseus* were used as an outgroup, but cropped from the final image. According to previous phylogenetic studies *Limnodromus* is the closest sister genus of *Limosa* [55]. RaxML [14] was used for the maximum likelihood analysis, with automated halting for bootstrap support.

## Results

### Microsatellite analysis (nuDNA)

A total of 132 different alleles were amplified. The number of alleles per locus ranged from 4 to 15, with no more than 2 alleles per individual. After sequential Bonferroni correction the breeding populations in The Netherlands showed a significant global heterozygote deficit at 6 loci, indicating low heterozygosity in this population. No significant linkage disequilibrium was found between any of the loci after sequential Bonferroni correction. MICROCHECKER detected no null alleles at any of the loci in the complete dataset.

For each sampling location, Table 2 reports the absolute number of alleles, allelic richness, F_IS_, and private alleles. Neither *L. l. islandica* nor *L. l. melanuroides* showed the presence of private alleles. F_IS_ values were significantly different from zero in The Netherlands and Belarus. AMOVA calculations showed significance for all the calculated variances. The molecular variance present in the sample set was explained for 3% by differences between sample locations. An additional 3% of the variance was explained by differences between individuals within locations. The remaining 94% was randomly distributed over populations, indicating the existence of genetic differentiation, although small, between populations. D supported differentiation between samples from Iceland and the other sampling locations (Table 3). Also, D indicated weak but significant differentiation between Dutch and Swedish samples (Table 3).

**Table 2:** Genetic diversity values of mitochondrial sequences and microsatellite fragment lengths.

Sample location/*L. limosa* subspecies	COI (n)	h	nh	HVR (n)	h	nh	Msats (n)	A	A_R_	P_a_	F_IS_
**Netherlands** *Limosa limosa limosa*	25	0.22	2	46	0.896	16	140	123	2.689	11	0.041^*^
**Mid-Germany** *Limosa limosa limosa*	8	0.25	2	9	0.972	8	23	84	2.673	0	−0.023
**Northern Germany** *Limosa limosa limosa*	4	0.00	1	4	1.000	4	12	70	2.582	0	0.072
**Denmark ** ***Limosa*** * limosa limosa*	3	0.00	1	4	0.833	3	11	68	2.579	1	0.002
**Belarus/Moscow** *Limosa limosa limosa*	4	0.00	1	6	1.000	4	6	55	2.581	0	0.189*
**Sweden ** ***Limosa*** * limosa limosa*	2	0.00	1	4	0.500	2	42	100	2.656	3	0.034
**Kazachstan/SW Russia** *Limosa limosa limosa*	4	0.40	2	8	0.929	6	23	97	2.695	4	−0.002
**Iceland** *Limosa limosa islandica*	3	0.00	1	5	0.900	4	27	62	2.355	0	0.054
**Eastern Russia** *Limosa limosa melanuroides*	1	na	Na	3	0.667	2	3	41	2.667	0	0.143

Sample location and *Limosa limosa* subspecies; number of sequence alignments (n), haplotype diversity (h), number of haplotypes (nh) for COI and HVR mtDNA; and number of individuals (n), absolute number of alleles (A), allelic richness (A_R_), number of private alleles (P_a_) and inbreeding coefficient (F_IS_) for microsatellite fragment analysis (Msats).

**Table 3:** D values for the microsatellite loci and pairwise Φst for mtDNA HVR sequences.

	Netherlands	M Germany	N Germany	Denmark	Belarus	Sweden	Kaz/W Rus	Iceland	E Russia
**Netherlands**	-	−0.03529	−0.06135	−0.04698	−0.05796	0.23901	−0.00394	0.53332*	0.91115*
**M Germany**	0.005	-	−0.13251	−0.11034	−0.09773	0.32468	−0.05243	0.51159	0.91407
**N Germany**	0.026	0.022	-	−0.21049	−0.11098	0.33619	−0.13143	0.47302	0.92038
**Denmark**	0.009	0.029	0.018	-	−0.06555	0.30287	−0.09829	0.44205	0.90956
**Belarus**	0.000	0.039	0.037	0.030	-	0.39894	−0.09067	0.51269	0.92515
**Sweden**	0.022*	−0.010	0.036	0.027	0.018	-	0.32384	0.59999	0.97015
**Kaz/SW Rus**	0.011	0.002	0.019	0.019	−0.000	0.017	-	0.54314	0.93078
**Iceland**	0.106*	0.088*	0.111*	0.094*	0.175*	0.134*	0.129*	-	0.90610
**E Russia**	−0.071	−0.061	−0.004	−0.040	−0.127	−0.042	−0.093	0.081	-

Below the diagonal: D values for the microsatellite loci; above the diagonal: pairwise Φst for mtDNA HVR sequences. Confidence Intervals not overlapping with zero for D values and significant P values after sequential bonferroni correction for Φst are indicated by *.

**Figure 2:**
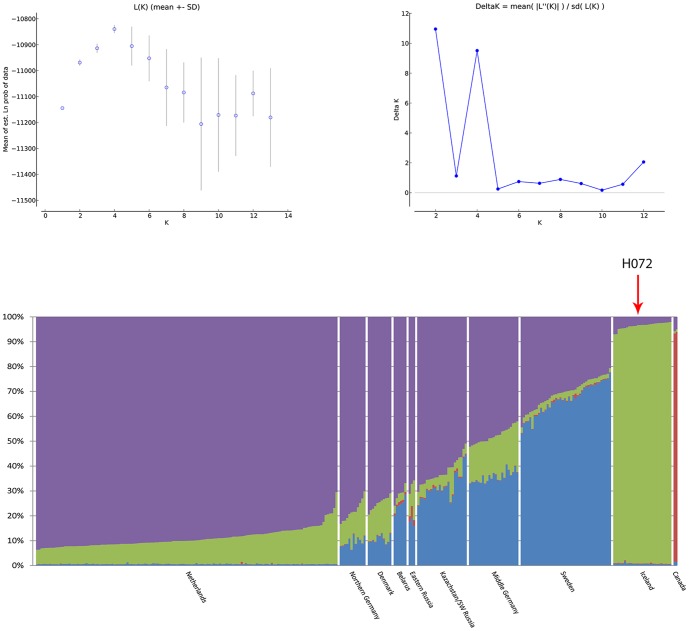
Mean log likelihood, DeltaK and assignment value plot of microsatellite STRUCTURE analysis. Above: mean log likelihood Ln P(X|K) and DeltaK as a function of the number of genetic clusters (K) averaged over 5 consecutive STRUCTURE runs for each K (error bars indicate one standard deviation). Below: representation of the assignment values, estimated relative contribution of each member of the population to that individual's microsatellite-based genome, per individual at the different sample locations for K = 4. The red arrow indicates sample H072.

Results from STRUCTURE strongly supported a scenario with four genetic groups. The maximum average log likelihood Ln P(X|K) showed a maximum at K = 4 (Figure 2). Birds from Iceland (*L. l. islandica*) and Canada (*L. heamstica*) were assigned to a separate cluster (group 3 and 4 respectively). Birds from the breeding range of *L. l. limosa* were assigned to two different genetic groups, hereafter groups 1 and 2 (Figure 2). Genotypes from individuals out of The Netherlands were assigned to group 1 almost completely. Assignment of the other *L. l. limosa* individuals was more ambiguous, with individuals of different sample locations being assigned mostly to group 1 or both groups 1 and 2. Only in the Swedish population did assignment of the genotypes to group 2 exceed 60% in most individuals. Eastern Russian birds (*L. l. melanuroides*) were not recognized as a distinct genetic entity, showing admixture of all groups. It is known that programs like STRUCTURE are very conservative in assigning samples from a certain group to a cluster when the sample sizes of such a group is small or sampling scheme is biased [53]. Therefore additional STRUCTURE analysis were preformed with pruned sets of three randomly chosen samples per sample location, for the entire dataset and for the dataset without *L. heamastica*. The analysis with the entire dataset only showed differentiation between *L. heamastica* and all other *Limosa* individuals according to maximum average log likelihood Ln P(X|K) (K = 2). The STRUCTURE analysis without the *L. heamastica* failed to detect any genetic groups (K = 1). This additional analysis, indicates that STRUCTURE is very sensitive to small sample size when having to assign individuals to genetic groups.

Mantel tests detected significant correlation between genetic distance and geographic distance (P = 0.006), but not when Icelandic birds were excluded (P = 0.313).

### Mitochondrial analysis (mtDNA)

**Figure 3:**
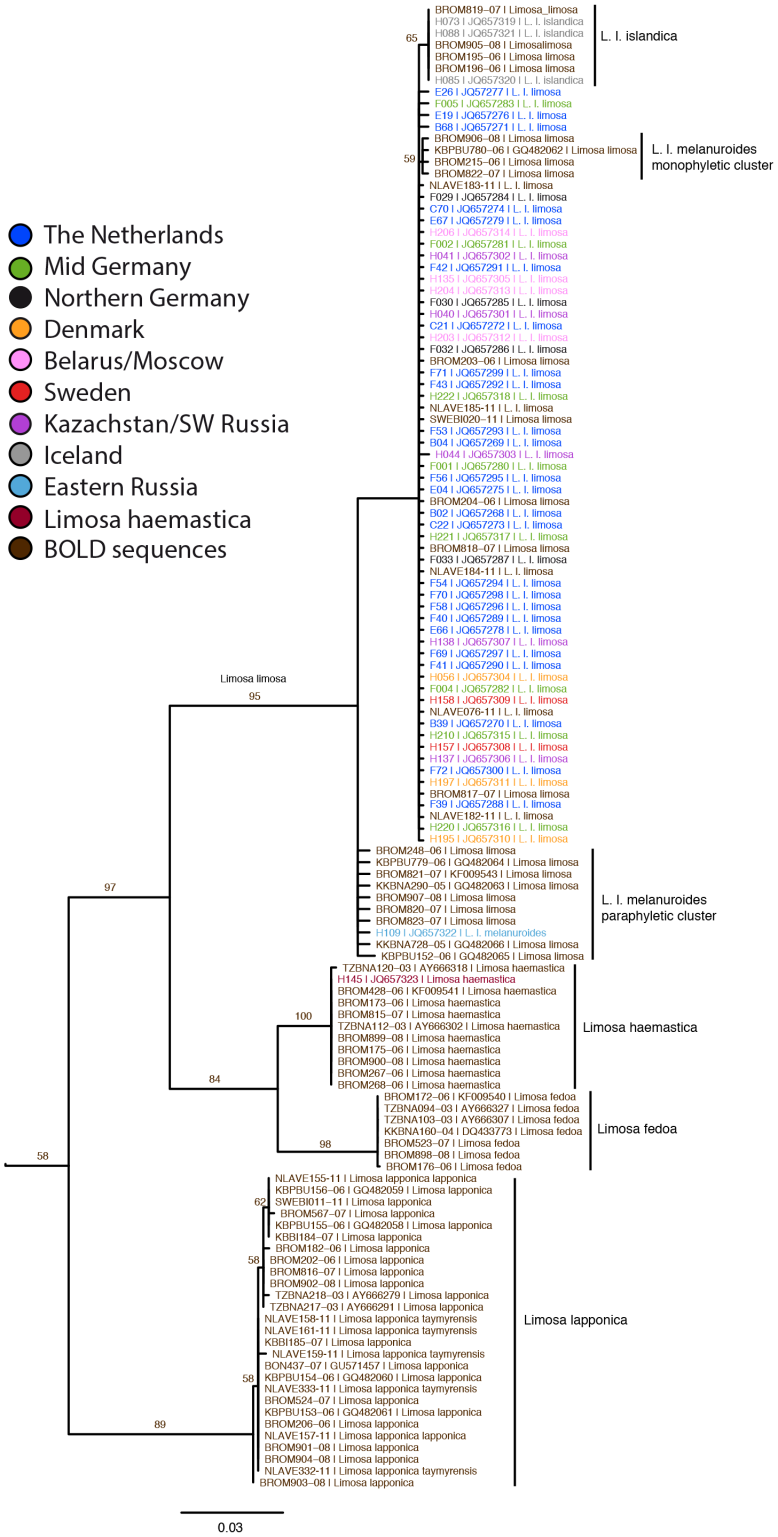
Maximum Likelihood tree mitochondrial COI region. Maximum Likelihood based on COI barcode mitochondrial sequences of *Limosa* with *Limnodromus* as outgroup [55]. Aside from the barcode sequences generated for this study, public sequences for *Limosa haemastica*, *Limosa fedoa*, *Limosa lapponica*, *Limnodromus scolopaceus* and *Limnodromus griseus* available through BOLD were included as well, indicated by their BOLD ID.

No NUMT issues could be detected in our COI sequences. The subset of COI barcode sequences from our dataset was combined with the public *Limosa* sequences on BOLD (Figure 3). Genetic distances between COI barcodes have been shown to be a good indicator of phylogenetic relationships [63]. In the COI tree, the clade containing *L. haemastica* and *L. fedoa* was the nearest sister to *L. limosa*, with 8.3% and 8.5% pairwise distance to each species, respectively. This makes them both appropriate as outgroup for the HVR phylogenetic analysis. *L. lapponica* was placed as sister to the above, with 10.4% pairwise distance to *L. limosa*. Within *L. l. limosa*, COI sequences were 100% identical for 57 individuals from samples throughout the breeding distribution of *L. l. limosa*. COI sequences were derived from different PCR batches, with samples from diverse sources including blood, eggshell and muscle tissue, from which DNA was extracted by different people and in different laboratory rooms. Moreover, all the public BOLD sequences also consisted of this most common haplotype. Lack of variation due to large-scale contamination issues can thus be ruled out. *L. l. islandica* sequences were placed within the *L. l. limosa* cluster, distinguished by a single diagnostic character. Our *L. l. melanuroides* sequence (H109) as well as several BOLD sequences formed a paraphyletic sister cluster to *L. l. limosa* and *L. l. islandica*, with minimally 2.0% pairwise distance. However, four BOLD sequences of specimens from the distribution range of *L. l. melanuroides* contained COI haplotypes that differed at a single position from the most common *L. l. limosa* haplotype and formed a monophyletic cluster. Three of these four specimens were collected in Vietnam and could therefore not be linked to a specific breeding location. However, one was collected at the Selanga river delta area (KBPBU780-06), which is a known *L. l. melanuroides* breeding area and the same location as our *L. l. melanuroides* samples.

**Figure 4:**
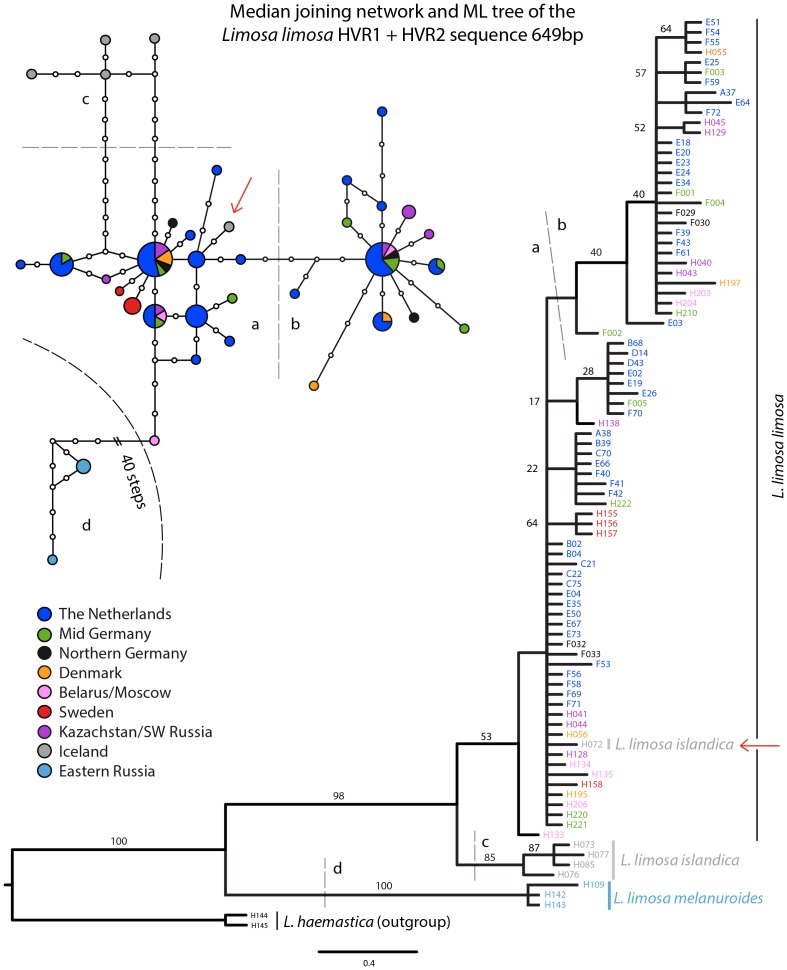
Maximum Likelihood tree and median joining network of the mitochondrial HVR1 and HVR2 regions. Analysis of the mitochondrial HVR sequences for the three *Limosa limosa* subspecies. The colors indicate the sample locations. The support values of the maximum likelihood analysis are plotted on the respective branches. Additionally, a median-joining network of 89 HVR mtDNA sequences is depicted. Different clusters are indicated with a/b/c/d. The red arrow indicates one individual (H072) which was found on Iceland but sorted close to *L. l. limosa* haplotypes.

Haplotype diversity (h) and number of haplotypes (nh) are summarized in Table 2. A total of 37 different haplotypes are found within the HVR dataset and in the COI dataset, within the genus *Limosa,* 7 different haplotypes were found. In the HVR dataset the number of variable sites was 117 and 7 indels were present. Phylogenetic trees of the mitochondrial HVR derived from the Maximum Likelihood analysis are shown in a Maximum Likelihood tree (Figure 4). Support values are displayed on the respective tree branches. Maximum Likelihood analyses support two monophyletic clades: one containing the individuals from Eastern Russia (bootstrap value 100%), the other containing all other individuals (bootstrap value 98%). The resolution of the HVR data was greater than that of COI barcode. All Icelandic samples but one were recovered on a monophyletic sister clade to the *L. l. limosa* clade, while a single sample from Iceland (H072) fell within the *L. l. limosa* clade, making *L. l. limosa* and *L. l*. *islandica* paraphyletic.

A median joining network based on the HVR sequences is shown in Figure 4. The basic structure of the network strongly resembles the phylogenetic tree but visualizes the relationships of the haplotypes within and between subspecies in a different way. Haplotypes of the individuals from Eastern Russia (d) are separated from all others (a/b/c) by at least 45 steps (Figure 4). Four Icelandic samples (c) are grouped together but separated from sample locations within the *L. l. limosa* breeding range (a/b) by at least 11 steps, while one Icelandic sample is found within the *L. l. limosa* cluster (red arrow in Figure 4). The individuals from the *L. l. limosa* breeding locations group into two star-shaped clusters (a/b), with the most common haplotypes separated by eight steps. The two star-shaped clusters do not correspond to geographically separated populations (Figure 4). Group haplotypes a and b were present evenly within al the sample locations (Netherlands, Mid and Northern Germany, Belarus, Denmark, Kazachstan/SW Siberia) of the *L. l. limosa,* except for Sweden where only group a haplotypes were found, as shown in the STRUCTURE analysis. Swedish *L. l. limosa* individuals belong to cluster a, but display two unique haplotypes. These results are supported by Φst calculations, which showed higher values for pairwise differences between Eastern Russian and all other individuals (Φst values between 0.91–0.97) as compared with pairwise differences between Icelandic and other individuals (Φst values between 0.44–0.54). Φst values between Sweden and other sample locations are moderate (Φst values between 0.24–0.40). Neither Fu's Fs (ranging from −9.47 to 1.61, P>0.50) nor Tajima's D (ranging from −1.32 to 1.32, P>0.10) nor Fu and Li's D* (ranging from −1.01 to 1.29, P>0.10) and F* (ranging from −0.95 to 1.32, P>0.10) are significant for the total population or any of the sampling locations.

## Discussion

Three subspecies have been recognized morphologically within *Limosa limosa* (*L. l. limosa*, *L. l. islandica* and *L. l. melanuroides*) and have been confirmed to be genetically identifiable as well in a previous study using the ‘conserved domain’ of the mitochondrial CR [23]. Here we confirm this distinction. Nevertheless, the signals found in the nuDNA did not support the split between *L. l. melanuroides* and *L. l. limosa* demonstrated by the mtDNA.

### Nuclear DNA

Nuclear DNA showed significant heterozygote deficiency in the Netherlands. MICROCHECKER analysis showed no signs of null alleles within this population, indicating that heterozygote deficiency was not an effect of null alleles. It is also unlikely that it was caused by a Wahlund effect [61]. As previous population genetic research could not detect any genetic population structure among Black-tailed Godwits breeding in different areas in The Netherlands [57], a possible explanation could be that there are few migration events from other locations towards The Netherlands (note significant F_IS_ value, Table 2).The nuDNA data demonstrates genetic differentiation between *L. l. islandica* on the one hand and *L. l. limosa* and *L. l. melanuroides* on the other. No genetic split between *L. l. melanuroides* and *L. l. limosa* was detected in the nuDNA. Two genetic groups could be detected within *L. l. limosa*. In most sample locations the genotype of the individuals is partitioned to the ‘purple’ genetic group (Figure 2); The Netherlands, Northern Germany, Denmark, Belarus, most individuals from Kazachstan/SW Russia and the *L. l. melanuroides* samples. The genotypes of the Black-tailed Godwit individuals from Sweden are mostly assign to the ‘blue’ genetic group. Individuals from Mid Germany show an admixture of genotypes between these three genetic groups.

### Mitochondrial DNA

Only three COI barcode haplotypes were found within *L. l. limosa*, 92% of all samples and showed the same haplotype. The *L.l. islandica* contained only a single haplotype where *L. l. melanuroides* showed two different haplotypes. The lack of subspecific variation in COI barcode has been noted for other bird species, too, with various explanations being given, including selective sweeps or genetic drift through population bottlenecks [26]. However, because the HVR data did contain variation, we suggest that for our case it is probably an artefact of the lower substitution rate in COI compared to the HVR region of the mtDNA [6], [62]. How the lower substitution rate for COI for birds compared to other groups might be explained is another matter. Even though the resolution exhibited by the COI barcode is less than the resolution of the HVR data, the subspecies are distinguishable by both parts of the mtDNA. *L. l. limosa* is divided into two large star-like haplotype clusters in the HVR median joining network. These clusters are not supported geographically, as both haplotype clusters are present at nearly all the *L. l. limosa* sample locations. The two *L. l. limosa* haplotype clusters in the HVR mtDNA (Figure 4; cluster a and b) do not completely correspond with the *L. l. limosa* genetic groups found in the nuDNA (Figure 2). Interestingly, both mtDNA regions (COI, HVR) show genetic differentiation between one *L. l. melanuroides* haplotype and *L. l. limosa* individuals to be much higher than that between *L. l. limosa* and *L. l. islandica* individuals. A single individual from Iceland (H072) contains a HVR haplotype that closely resembles that of *L. l. limosa* individuals. To confirm that this was not due to contamination, we re-examined the microsatellite results from this extract. The microsatellite genotype of H072 was unique and contamination of the extract was thus ruled out; the lowest genetic distance found in all pairwise comparisons with H072 was 8 differences. Furthermore, we repeated the HVR PCR and sequencing for this sample twice, with no change in the results. This could have been caused by a misidentification of a *L. l. limosa* individual as a *L. l. islandica*. While this individual was caught on its nest in Iceland which is a location believed to harbour breeding *L. l. islandica* only, a recently published paper demonstrates the overlap of migration routes of *L. l. islandica* and *L. l. limosa*, and advocate that current overlap in breeding areas is also possible [31]. Furthermore, they demonstrate that identifying *L. l. limosa* individuals from *L. l. islandica* individuals based purely on morphological differences sometimes fails, due to the highly polymorphic nature of Black-tailed Godwits [31]. If H072 was indeed misidentified this would mean that *L. l. limosa* individuals are breeding at *L. l. islandica* breeding location and might even hybridize with *L. l. islandica* individuals. The fact that H072 was not partitioned in the *L. l. limosa* cluster in the STRUCTURE analysis suggests that there has been a *L. l. limosa* female dispersal event towards Iceland.

### nuDNA vs mtDNA: *L. l. islandica*

The differentiation between *L. l. limosa* and *L. l. islandica* shows similar patterns in the mtDNA and nuDNA. Within the mtDNA private haplotypes in *L. l. islandica* do not support a scenario of mitochondrial gene flow between *L. l. limosa* and *L. l. islandica*. Furthermore, *L. l. islandica* does not possess private nuclear alleles but differs from *L. l. limosa* only by its allele frequencies. Together, the nuDNA and mtDNA thus suggest relatively recent separation of *L. l. islandica* and *L. l. limosa*.

### nuDNA vs mtDNA: *L. l. melanuroides*

While the differentiation between *L. l. limosa* and *L. l. islandica* shows similar patterns in mtDNA and nuDNA, differentiation between *L. l. limosa* and *L. l. melanuroides* seems to show opposite patterns in the mtDNA and nuDNA. The HVR part of the and the COI paraphyletic cluster in the mtDNA exhibited a sharp divergence between *L. l. melanuroides* and the remaining Black-tailed Godwits, while in the nuDNA there was a lack of divergence between *L. l. melanuroides* and *L. l. limosa*. As STRUCTURE analysis of pruned datasets showed, these results can most likely be explained by the low sample size of *L. l. melanuroides* which has probably obscured the genetic signal of a split between *L. l. melanuroides* and *L. l. limosa*. A recent study supports the results of this study in regards to the presence of two COI *L. l. melanuroides* haplotype groups, one paraphyletic cluster basal to *L. l. limosa* but showing a differentiation *with L. l. limosa* and one monophyletic cluster showing less distinct divergence from *L. l. limosa*, at the Selanga River Delta area [11]. This suggests that two different split events took place at this location and that these groups are still present as two disjunct but different *L. l. melanuroides* breeding colonies at this location. Misidentification could explain these results partly as well. Misidentification of *L. l. limosa* individuals as *L. l. melanuroides* is not very likely for the paraphyletic *L. l. melanuroides* group in COI as individuals from this group showed some divergence with *L. l. limosa* in COI and H109 a sample of the paraphyletic group in COI showed high divergence with *L. l. limosa* in the HVR. The *L. l. melanuroides* individuals of the monophyletic cluster in the COI tree could in theory have been misidentified, although this is very unlikely since the four samples were taken at two different locations. While, *L. l. melanuroides* are smaller than *L. l. limosa* and migration routes are largely separated some overlap in morphology and migration might still exist. If indeed these individuals were all misidentified then this would implicate that at the Selanga River Delta area *L. l. melanuroides* and *L. l. limosa* are breeding in close proximity of each other.

### nuDNA vs mtDNA: within *L. l. limosa*

While the mtDNA demonstrated that haplotypes belonging to both cluster a and b were grossly present in all sample locations, the nuDNA shows that the genotypes of the *L. l. limosa* individuals from the Netherlands, Northern Germany, Denmark, Belarus, and most individuals from Kazachstan/SW Russia are assigned mostly to one genetic group and the bigger part of the genotypes of most Swedish individuals to another genetic group. As the HVR mtDNA shows that structure within *L. l. limosa* is more recent than the divergence with *L. l. islandica*, one explanation for the different *L. l. limosa* patterns in mtDNA and nuDNA might be incomplete lineage sorting in the microsatellites. Alternatively, northward founder events by two separate *L. l. limosa* lineages subsequently expanding throughout the current *L. l. limosa* breeding range, genetically homogenizing the historically present *L. l. limosa* breeding populations found in the nuDNA. This event in turn could have been followed by recent isolation and genetic drift which would explain the two distinct star-shaped HVR mtDNA haplotype clusters for *L. l. limosa* (a and b) and the three genetic groups present in the nuDNA. Similar patterns have been found in the Herring Gull *Larus argentatus* complex [30]. Some divergence between Sweden and other *L. l. limosa* sampling locations is shown by the the STRUCTURE analysis. Additionally, D estimates showed very weak differentiation between Sweden and The Netherlands. Whilst the Swedish *L. l. limosa* individuals do not share any mtDNA haplotypes with other *L. l. limosa* individuals, they are closely related to other *L. l. limosa* individuals, which might indicate recently restricted gene flow between Swedish *L. l. limosa* and other *L. l. limosa* individuals.

### Molecular dating of splits

Wenink and Baker [62] and Buehler and Baker [6] estimated the mutation rates for HVR1 and HVR2 at around 10% per Myr. For a sequence length of 649 bp this would translate to 6.4×10^−5^ mutations per year, with a range of 3.2×10^−5^ to 9.6×10^−5^. This results in split estimates of approximately 347 (±174) Ky for *L. l. limosa* vs. *L. l. melanuroides* (45 mutations), 85 (±43) Ky for *L. l. limosa* vs. *L. l. islandica* (11 mutations) and 62 (±31) Ky for the two mtDNA *L. l. limosa* (8 mutations) clusters. This would indicate that the mtDNA population structure, according to HVR, arose during the Pleistocene. Other studies have also reported the origin of lineage diversity of several bird species to lie within the Pleistocene [24], [36], [45]. Iceland was covered in ice during the Weichselien (occuring between 116Ky – 11,5Ky), making it unlikely that *L. l. islandica* (85Ky ago) colonized the island during that period [1], [52]. We hypothesize that the most recent common ancestor of *L. l. islandica* colonized Iceland after the Pleistocene (i.e. in the last 12Ky) and that since then genetic isolation and drift have resulted in the genetic differentiation observed between these subspecies today. Lineage diversification between *L. l. limosa* and *L. l. melanuroides* lineages could have occurred via separate southward or northward founder events. During the Pleistocene the ice sheets that dominated the landscape in Northern Europe and America were absent in large parts of far eastern Russia and there is strong evidence from Beringia and north-eastern Asia that several species of plant and animal survived the last glaciation at high altitudes [1], [52]. We suggest that the ancestral *L. l. melanuroides* became isolated from the remaining Black-tailed Godwit population at different times in the Beringian refugium during periods of glacial cooling in the Pleistocene, resulting in the two splits in the mtDNA.

### Conclusions

Our data confirm divergence between the three *Limosa limosa* subspecies. According to the patterns observed and their geographic separation, we propose that the three traditional subspecies should be managed as three separate units. However, our data do indicate that *L. l. limosa* individuals might have bred between *L. l. islandica* individuals at Iceland recently. We believe the most likely explanation for the genetic structure found in this study is post-Pleistocene geographical separation of *L. l. islandica*, and at least one *L. l. melanuroides* group and a distant Pleistocene split of another *L. l. melanuroides* group. The two star-shaped haplotype clusters visible in the mtDNA of *L. l. limosa* are most likely the result of one or more successful *L. l. limosa* populations carrying two ancestral haplotypes expanding post-Pleistocene throughout the current *L. l. limosa* breeding range. Our data highlight the importance of using both nuDNA and mtDNA simultaneously when studying range-wide population genetic structure in birds.
